# Identification of differential splicing genes in gliomas using exon expression profiling

**DOI:** 10.3892/mmr.2014.2775

**Published:** 2014-10-27

**Authors:** FENG YU, WEI-MING FU

**Affiliations:** Department of Neurosurgery, The Second Affiliated Hospital, College of Medicine, Zhejiang University, Hangzhou, Zhejiang 310009, P.R. China

**Keywords:** glioblastomas, oligodendrogliomas, alternative splicing, differentially expressed genes, protein-protein interaction network

## Abstract

Diffuse gliomas are the most common type of malignant primary brain tumor, and their initiation and/or progression are often associated with alternative splicing. They produce an enormous economic burden on society and greatly impair the quality of life of those affected. The aim of the current study was to explore the differentially expressed genes (DEGs) observed in glioblastoma (GBM) and oligodendroglioma (OD) at the splicing level, and to analyze their functions in order to identify the underlying molecular mechanisms of gliomas. The exon-level expression profile data GSE9385 was downloaded from the Gene Expression Omnibus database, and included 26 GBM samples, 22 OD samples and 6 control brain samples. The differentially expressed exon-level probes were analyzed using the microarray detection of alternative splicing algorithm combined with the splicing index method, and the corresponding DEGs were identified. Next, a Gene Ontology enrichment analysis of the DEGs was performed. Additionally, the protein-protein interaction (PPI) networks were constructed based on the depth-first search algorithm. A total of 300 DEGs were identified to be shared by GBM and OD, including 97 upregulated and 203 downregulated DEGs. Furthermore, screening with a defined threshold identified 6 genes that were highly expressed in GBM, including *AFF2*, *CACNA2D3* and *ARPP21*, while the 6 highly expressed genes in OD notably included *CNTN2*. The *TP53* and *HIST1H3A* genes were the hub nodes in the PPI network of DEGs from GBM, while *CNTN2* was linked to the highest degree in the OD PPI network. The present study provides a comprehensive bioinformatics analysis of DEGs in GBM and OD, which may provide a basis for understanding the initiation and/or progression of glioma development.

## Introduction

Diffuse gliomas are the most common type of intracranial malignant neoplasm, and account for >60% of all primary brain tumors (1). Based on the classification of nervous system tumors by the World Health Organization, diffuse gliomas are classified into seven principal categories: Diffuse astrocytoma (grade II), oligodendroglioma (OD; grade II), oligoastrocytoma (grade II), anaplastic astrocytoma (grade III), anaplastic oligodendroglioma (grade III), anaplastic oligoastrocytoma (grade III) and glioblastoma (GBM, grade IV) (1). Of these, GBM is the most common and aggressive type of primary brain tumor, accounting for 80% of malignant astrocytomas (2). GBM may develop rapidly without the diagnosis of a less malignant precursor lesion, and this is termed primary or *de novo* GBM. It may also develop slowly through progression from a pre-existing low-grade glioma, in which case it is termed a secondary GBM (3). Although GBMs are considered as primarily astrocytic gliomas (4), a subset of GBMs exhibit OD-like tumor cell differentiation (5). OD is a well-differentiated, slowly grown and diffusely infiltrated tumor observed in adults, and is typically located in the cerebral hemispheres (6). Despite advances in neurosurgery, chemotherapy and radiotherapy, glioma commonly has a poor prognosis (7). Therefore, it is critical that the genetic pathways underlying the development of this type of cancer are defined.

A previous study indicated that tumor-specific alternative splicing is important in the regulation of gene expression and corresponding protein functions during cancer development (8). Multiple alternative splicing transcripts have been identified as progression markers, including generalized splicing abnormalities and tumor- and stage-specific events (9–10). A number of studies have documented that the initiation and/or progression of glial brain tumors is influenced by aberrant splice isoforms, including epidermal growth factor receptor, phosphatase and tensin homolog, tumor protein p53 (*TP53*), proliferation-related Ki-67 antigen, murine double-minute 2, mutS homolog 2, platelet-derived growth factor α and Kruppel-like transcription factor (11–15). However, the molecular mechanisms associated with the alternative splicing that may lead to the development and progression of GBM and OD remain to be clearly demonstrated.

In the current study, the gene expression profiles of GBM, OD and patient-matched normal brain tissues were downloaded from the Gene Expression Omnibus (GEO) database. The significant differentially expressed exon-level probes and their corresponding genes were identified using a combination of the splicing index (SI) method and the microarray detection of alternative splicing (MIDAS) algorithm. In addition, the screened differentially expressed genes (DEGs) were further analyzed with bioinformatics methods. The current study aims to improve the understanding of molecular mechanisms of GBM and OD and may clarify the processes involved in the development of gliomas.

## Materials and methods

### Affymetrix microarray analysis

The gene expression profile data GSE9385 (7) was obtained from the National Center for Biotechnology Information and GEO database (http://www.ncbi.nlm.nih.gov/geo/), which is based on the GeneChip Human Exon 1.0 ST Array (GPL5188) platform (Affymetrix, Santa Clara, CA, USA). A total of 54 specimens were available, including 26 GBM, 22 OD and 6 control brain samples.

### Data preprocessing

The background correction and data normalization were performed by the robust multiarray average (RMA) algorithm based on the Affymetrix Power Tools (http://www.affymetrix.com/) program (16). Additionally, the probe sets were filtered according to the methods described by Gardina *et al* (17). To reduce the false positive rate, only the genes with a PLIER signal of >200 and corresponding detection above background (DABG) with a P-value of ≤0.05 were accepted. Probe sets with cross-hybridization type were also removed (18).

### DEG analysis

The differences in exon-level expression can result from one of two factors, namely, differential splicing or differential gene expression (18). To detect differential splicing, the gene expression level was normalized. Following normalization, the exon-level expression value (I) was calculated for each exon to reflect the actual exon-level expression. For exon *i* in gene *j*, I*_i,j_* is denoted as follows:

$$I_{i,j}=\frac{E_{i,j}}{G_{j}}\cdot G$$

G is the average gene expression value of all specimens and G*_j_* is the gene expression value of specimen *j*. E*_i,j_* represents the expression values of exon *i* in gene *j*. Analysis using this formula is known as the SI method (18,19). The exon- and gene-level expression values were computed using the RMA algorithm.

Based on the SI method, the differentially expressed exon-level probes between GBM/OD, GBM/normal and OD/normal samples were identified with Student’s t-test. Only exon-level probes with P-values <0.01 and differential regulation of RMA signals >2-fold were selected. Since the RMA-generated signals were reported as the value of log2 transformation, the geometric mean >1 represented an RMA signal that was upregulated >2-fold. Also, in order to improve the accuracy of the results, the MIDAS algorithm (http://www.affymetrix.com/) (17) was used to identify differentially expressed exon-level probes based on the analysis of variance. P<0.05 was selected as the cut-off criterion. Only the exons identified by the two methods were used. A hierarchical clustering of the screened DEGs was performed based on their expression values using the Hclust package of R software (20).

### Gene ontology (GO) enrichment analysis

GO analysis is a commonly used approach for functional studies of large-scale genomics (21). The Database for Annotation, Visualization and Integrated Discovery (DAVID), a high-throughput and integrated data-mining environment, analyzes gene lists derived from high-throughput genomic experiments (22). The current study used the DAVID to identify which represented GO categories were significantly enriched in DEGs.

### Protein-protein interaction (PPI) network construction

The Search Tool for the Retrieval of Interacting Genes (STRING) (23) database was used to annotate functional interactions between differentially expressed proteins that regulate alternative splicing and other proteins by calculating their confidence score. Interactions with a score >0.8 were selected. In order to remove the irrelevant nodes, reduce the network size and restrict the search space into a realistic and meaningful one, the depth first search algorithm was employed to construct the PPI network with the path length <3.

## Results

### Identification of DEGs

Exon-level expression data were compared between GBM/OD samples, GBM/normal samples and OD/normal samples. Using the SI method, 1,343 differentially expressed exon-level probes with P-values <0.01 and differential regulation of RMA signals >2-fold were identified. Based on the MIDAS method, 5,290 differentially expressed exon-level probes with the P-value <0.05 were selected. A total of 982 DEGs were identified by both methods simultaneously.

**Figure 1:**
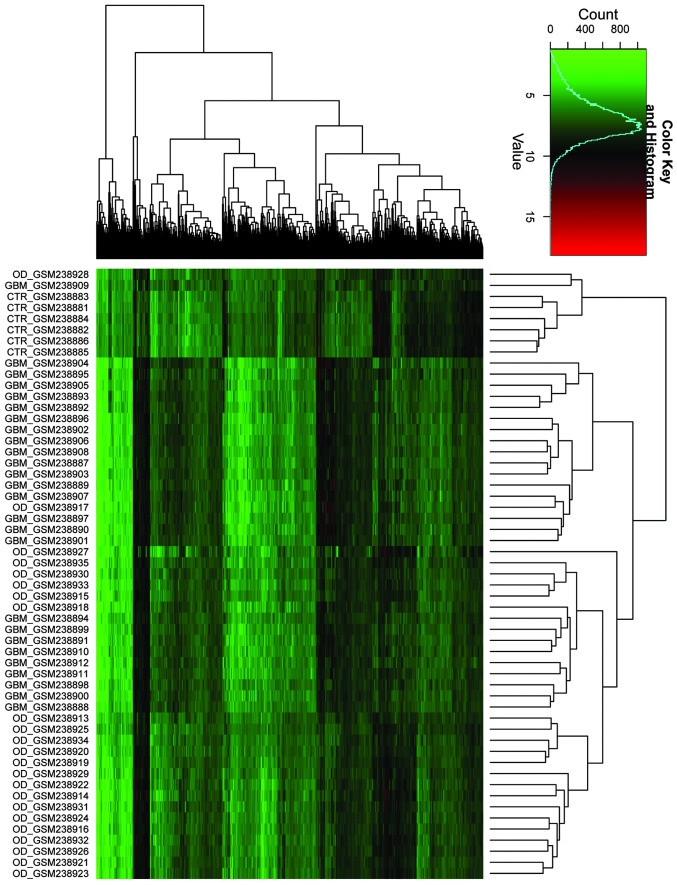
Hierarchical cluster analysis of 982 differentially expressed genes by the hclust package of R software.

**Figure 2:**
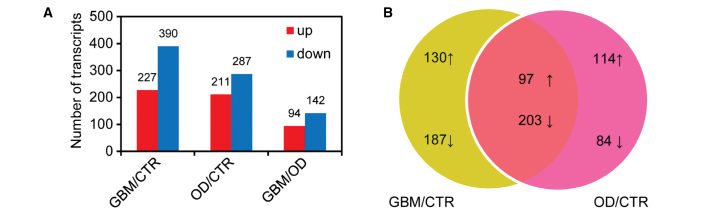
Identification of DEGs using the SI method and MIDAS algorithm. (A) DEGs between GBM/OD samples, GBM/normal samples and OD/normal samples. (B) A total of 617 and 498 differentially expressed exon-level genes were identified in GBM and OD compared with the control, respectively. A total of 97 upregulated and 203 downregulated genes were identified in both GBM and OD. DEG, differentially expressed gene; SI, splicing index; MIDAS, microarray detection of alternative splicing; GBM, glioblastoma; OD, oligodendroglioma; CTR, control.

The expression values of these 982 DEGs were hierarchically clustered by the Hclust package of R software (Fig. 1). Compared with normal brain tissue, 617 and 498 DEGs were identified in GBM and OD, respectively (Fig. 2A). A total of 97 upregulated and 203 downregulated DEGs were identified to be present in both GBM and OD (Fig. 2B), and 236 DEGs were obtained between GBM and OD, 94 of which were upregulated and 142 downregulated in GBM (Fig. 2A).

With the strict threshold of RMA signals that were upregulated at least 4-fold and 2-fold compared with normal brain tissue and OD respectively, a total of 6 highly expressed genes were identified in GBM, including *AFF2*, *GNAL*, *ARPP21*, *CACNA2D3*, *HIST1H3A*~*HIST1H3J*, and *RGS7* (Table I). Similarly, at the cut-off criteria of RMA signals upregulated at least 4-fold and 2-fold compared with normal brain tissue and GBM respectively, a total of 6 highly expressed genes were identified in OD, including *CNTN2*, *ABCA6*, *MEGF11*, *DOCK5*, *MOXD1*, and *TRIM67*. There were 7 genes downregulated at least 8-fold compared with controls in both GBM and OD. These included *APBA2*, *MAP4, NUF2, INPP5F* and *TOP2A*. These candidate genes were suggested as markers of alternative splicing in GBM and OD.

### GO enrichment analysis

To investigate the functional changes in the initiation and/or progression of glial brain tumors, the 300 overlapping DEGs in GBM and OD were mapped to the GO database. A P-value ≤0.01 and fold change >2 were used for the threshold. The significant GO terms of the 97 upregulated DEGs shared by GBM and OD included various processes, including cell adhesion, extracellular structure organization, collagen biosynthetic process, neuron development and the regulation of small GTPase-mediated signal transduction (Table II). For the 203 downregulated DEGs shared by GBM and OD, the significant GO terms included processes such as the regulation of small GTPase-mediated signal transduction, transmission of nerve impulses and positive regulation of apoptosis (Table II).

The DEGs that were upregulated ≥2-fold in one subgroup compared with the other were also mapped to the GO database. A total of 94 upregulated DEGs in GBM were significantly enriched into 10 GO terms, including neuron differentiation, exocytosis and regulation of neurotransmitter secretion (Table III). Additionally, 42 upregulated DEGs in OD were significantly enriched into 7 GO terms, which included the transmission of nerve impulses, cell-cell signaling and synaptic transmission (Table IV).

### PPI network construction

**Figure 3:**
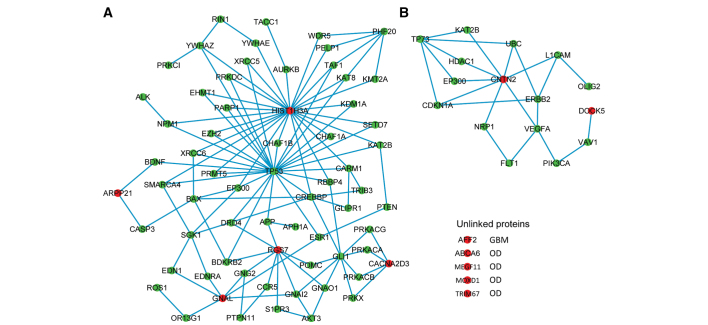
Protein-protein interaction network of highly expressed genes in (A) glioblastoma and (B) oligodendroglioma.

In order to construct the PPI network, the depth-first search algorithm was employed to obtain the PPI data from the STRING database. PPI networks of highly expressed genes in GBM (Fig. 3A) and OD (Fig. 3B) were constructed with the path length <3. In the PPI network of GBM, the genes *HIST1H3A* and *TP53* contained the highest degrees. In addition, in the PPI network of OD, *GNTN2* acted as hub nodes.

## Discussion

Formation and malignant progression of diffuse gliomas are associated with alterations in a variety of genes that regulate the normal homeostasis of cell proliferation, differentiation and apoptosis (24). The connection between abnormal regulation of alternative splicing and tumor development has emerged as a novel aspect of cancer biology. The identification of alternatively spliced genes in GBM and OD may provide novel molecular markers for the diagnosis and treatment of the two subtypes of glioma.

In the present study, 300 overlapping DEGs were identified in GBM and OD, compared with normal control tissue. These included 97 upregulated and 203 downregulated DEGs. Notably, 117 of these 300 DEGs were associated with alternative splicing. With the strict threshold, 6 highly expressed genes were screened in GBM, including *AFF2*, *GNAL*, *ARPP21*, *CACNA2D3* and *RGS7*, in addition to 6 highly expressed genes in OD, including *CNTN2*, *ABCA6*, *MEGF11*, *DOCK5*, *MOXD1* and *TRIM67*. Finally, by constructing a PPI network of DEGs, it was demonstrated that *TP53* and *HIST1H3A* were the hub nodes in the PPI network of GBM and *CNTN2* was the hub node in OD.

*AFF2*/*FMR2* is extended over >600 kb in Xq27.3–q28, composed of 22 exons with a complex pattern of alternative splicing (25). It has been demonstrated that *AFF2* mutations are associated with breast tumors (26). Recently, an excess of non-synonymous missense variants in *FMR2* has been reported in males with autism spectrum disorders (27), indicating the role of *FMR2* in normal brain function. The silencing of the *AFF2* gene can lead to Fragile XE syndrome (28). In agreement with a previous study, the current study indicated that the *AFF2* gene with differentially spliced exons was highly expressed in GBM. Bensaid *et al* (29) reported that as an RNA-binding protein, the *AFF2* protein serves an essential role in alternative splicing regulation via the interaction with the G-quartet RNA-forming structure. *CACNA2D3* protein is an auxiliary member of the α-2/δ subunit family of the voltage-dependent calcium channel complex (30). The *CACNA2D3* gene has been suggested as a putative tumor suppressor gene in lung cancer, renal cell cancer neuroblastoma and squamous cell esophageal cancer (31), and has been identified as an indicator of prognosis in gastric cancer (32). *CACNA2D3* is highly expressed in neuroblasts and neuroblastomas with a favorable prognosis, while its expression is downregulated in those with a poor prognosis (33). Additionally, *ARPP21* encodes a 21-kDa cAMP-regulated phosphoprotein termed regulator of calmodulin signaling, which is enriched in the brain and may serve as a candidate tumor suppressor gene (34). *ARPP21* is frequently downregulated, as is miR-128-2, in human breast cancer (35,36). Notably, miR-128 expression may significantly reduce glioma cell proliferation *in vitro* and glioma xenograft growth *in vivo* (37). In addition, Donzelli *et al* (36) reported that mutant *TP53* is able to bind the putative promoter of the miR128-2 host gene (*ARPP2*), which determines the concomitant induction of *ARPP21* mRNA expression in lung cancer cells, and thus inhibits apoptosis (36). In the present study, *TP53* acted as the hub node in the GBM network, indicating its important role in the initiation and/or progression of GBM. A previous study suggested that *TP53* regulates the proliferation, differentiation and survival of stem cells, which further highlights the importance of *TP53* in GBM suppression (38). Additionally, *TP53* mutations have been noted in 5–15% of cases of OD (39). *TP53* mutations are the most frequent type of gene-specific alteration identified in human cancers (40). Shiraishi *et al* (41) studied the different locations of *TP53* mutations between anaplastic astrocytoma and GBM, and suggested that the *TP53* mutation may contribute to tumorigenesis and also to the progression of malignancy in gliomas.

It has been suggested that *CNTN2* (also knows as contactin-2 or axonal glycoprotein TAG-1) is involved in the cell adhesion process and serves a critical function in the early stages of hepatocellular carcinoma (42). Adair *et al* (43) have also demonstrated that the *CNTN2* gene is expressed in a variety of tumor cell lines, including those from the brain, breast and lung, and particularly in an unusually high percentage of melanoma cells. In the present study, *CNTN2,* with a high expression level, was the hub node in the PPI network of OD, suggesting that it may serve an essential function in the pathogenesis of OD.

GO enrichment analysis indicated a close relationship between the two subsets of gliomas and cell adhesion, which is in agreement with previous studies (44,45). An essential step in tumor progression is tumor invasion, which is dependent on the preservation of a delicate balance between cell adhesion and cell detachment. Chen *et al* (42) have suggested that the cell adhesion pathway is important for cancer cell invasion and metastasis. Abnormality of cell adhesion pathways is considered as a characteristic of advanced cancer. Another study demonstrated that alterations in several classes of adhesion molecules were implicated in the progression of various forms of cancer, including GBM (46).

In conclusion, the current data provide a comprehensive bioinformatics analysis of the alternatively spliced genes that may be involved in the pathogenesis of GBM and OD. A total of 300 overlapping DEGs were identified between GBM and OD. In addition, compared with normal brain tissue and OD, 6 highly expressed genes were identified in GBM, while 6 were identified in OD compared with normal brain tissue and GBM. The present analysis provides a basis for the understanding of the molecular mechanism of GBM and OD. However, further experimental studies with larger sample sizes are required to confirm these observations.

## Figures and Tables

**Table I:** Identification of differentially expressed genes between GBM/OD samples, GBM/normal samples and OD/normal samples.

		GBM/CTR		OD/CTR		GBM/OD	
				
Type	Gene name	log2 transformation	P-value	log2 transformation	P-value	log2 transformation	P-value
GBM	*AFF2*	2.18	2.86E-03	−0.36	5.66E-01	2.55	2.48E-07
GBM	*GNAL*	2.46	2.08E-05	0.73	8.30E-02	1.74	3.74E-07
GBM	*ARPP21*	2.75	3.26E-07	1.1	4.11E-02	1.65	2.85E-06
GBM	*CACNA2D3*	3.37	1.10E-09	1.76	4.31E-05	1.61	1.98E-05
GBM	*HIST1H3A*~*HIST1H3J*	2.91	1.92E-08	1.48	8.97E-04	1.43	3.67E-07
GBM	*RGS7*	3.36	3.08E-07	1.97	9.74E-05	1.39	1.17E-04
OD	*CNTN2*	1.72	2.84E-03	2.84	1.40E-07	−1.11	1.22E-03
OD	*ABCA6*	1.42	6.40E-03	2.6	1.38E-04	−1.18	1.00E-03
OD	*MEGF11*	1.11	1.37E-02	2.26	1.17E-06	−1.15	1.78E-05
OD	*DOCK5*	0.91	2.19E-02	2.03	6.19E-05	−1.12	1.59E-04
OD	*MOXD1*	−0.24	4.79E-01	2.45	2.01E-05	−2.69	5.29E-10
OD	*TRIM67*	−0.37	6.15E-01	2.54	1.62E-04	−2.91	9.58E-09
GBM + OD	*APBA2*	−4.49	1.89E-08	−3.12	1.93E-05	−1.36	1.86E-03
GBM + OD	*MAP4*	−4.1	1.69E-08	−3.13	3.54E-06	−0.97	1.17E-02
GBM + OD	*NUF2*	−3.98	1.86E-09	−3.16	3.67E-05	−0.82	3.31E-02
GBM + OD	*INPP5F*	−3.17	2.41E-09	−3.33	1.64E-09	0.16	5.27E-01
GBM + OD	*TOP2A*	−8.68	4.11E-15	−7.87	4.60E-08	−0.81	1.85E-01

GBM, glioblastoma; OD, oligodendroglioma; CTR, control.

**Table II:** Significant GO terms of 97 upregulated and 203 downregulated DEGs shared by GBM and OD.

Terms	Description	Count	P-value	FDR
Upregulated DEGs				
GO:0007155	Cell adhesion	15	2.02E-06	3.06E-03
GO:0043062	Extracellular structure organization	7	8.96E-05	0.14
GO:0032964	Collagen biosynthetic process	3	1.98E-04	0.30
GO:0048666	Neuron development	8	7.96E-04	1.20
GO:0051056	Regulation of small GTPase-mediated signal transduction	7	9.28E-04	1.40
GO:0031175	Neuron projection development	7	1.01E-03	1.51
GO:0030030	Cell projection organization	8	1.28E-03	1.92
GO:0008624	Induction of apoptosis by extracellular signals	5	1.62E-03	2.42
GO:0048667	Cell morphogenesis involved in neuron differentiation	6	2.47E-03	3.68
GO:0048812	Neuron projection morphogenesis	6	2.69E-03	3.98
GO:0008088	Axon cargo transport	3	2.92E-03	4.33
GO:0043065	Positive regulation of apoptosis	8	3.10E-03	4.58
GO:0030182	Neuron differentiation	8	3.43E-03	5.06
GO:0000904	Cell morphogenesis involved in differentiation	6	4.79E-03	7.01
GO:0048858	Cell projection morphogenesis	6	4.88E-03	7.12
GO:0000902	Cell morphogenesis	7	5.23E-03	7.62
GO:0032990	Cell part morphogenesis	6	5.86E-03	8.50
Downregulated DEGs				
GO:0051056	Regulation of small GTPase-mediated signal transduction	11	2.57E-05	0.04
GO:0019226	Transmission of nerve impulses	12	8.44E-05	0.13
GO:0043065	Positive regulation of apoptosis	11	1.85E-03	2.92
GO:0007268	Synaptic transmission	9	2.22E-03	3.49
GO:0007267	Cell-cell signaling	13	2.34E-03	3.68
GO:0001662	Behavioral fear response	3	5.87E-03	8.978
GO:0046578	Regulation of Ras protein signal transduction	7	5.87E-03	8.98

GO, gene ontology; DEG, differentially expressed gene; GBM, glioblastoma; OD, oligodendroglioma; FDR, false discovery rate.

**Table III:** Significant GO terms of DEGs in GBM that were upregulated at least 2-fold compared with OD.

Term	Description	Count	P-value	FDR
GO:0030182	Neuron differentiation	9	2.44E-04	0.36
GO:0006887	Exocytosis	5	9.80E-04	1.44
GO:0046928	Regulation of neurotransmitter secretion	3	2.90E-03	4.21
GO:0031175	Neuron projection development	6	2.94E-03	4.25
GO:0006904	Vesicle docking during exocytosis	3	3.79E-03	5.45
GO:0048278	Vesicle docking	3	4.44E-03	6.36
GO:0051588	Regulation of neurotransmitter transport	3	4.44E-03	6.36
GO:0060627	Regulation of vesicle-mediated transport	4	5.95E-03	8.44
GO:0016192	Vesicle-mediated transport	8	6.20E-03	8.78
GO:0022406	Membrane docking	3	6.28E-03	8.88

GO, gene ontology; DEG, differentially expressed gene; GBM, glioblastoma; OD, oligodendroglioma; FDR, false discovery rate.

**Table IV:** Significant GO terms of DEGs in OD that were upregulated at least 2-fold compared with GBM.

Term	Description	Count	P-value	FDR
GO:0019226	Transmission of nerve impulses	11	8.06E-05	0.12
GO:0007267	Cell-cell signaling	14	1.13E-04	0.17
GO:0007268	Synaptic transmission	10	1.23E-04	0.19
GO:0006836	Neurotransmitter transport	6	1.92E-04	0.29
GO:0007016	Cytoskeletal anchoring at plasma membrane	3	1.12E-03	1.69
GO:0007269	Neurotransmitter secretion	4	1.33E-03	2.01
GO:0003001	Generation of a signal involved in cell-cell signaling	5	2.17E-03	3.26

GO, gene ontology; DEG, differentially expressed gene; GBM, glioblastoma; OD, oligodendroglioma; FDR, false discovery rate.
